# Supplementary material for: IL-7Rα glutamylation and activation of transcription factor Sall3 promote group 3 ILC development
Source: Nat Commun. 2017 Aug 10;8:231. doi: 10.1038/s41467-017-00235-x (PMC5550436; doi:10.1038/s41467-017-00235-x)
Supplement: Supplementary file 1 — Supplementary Information [file 41467_2017_235_MOESM1_ESM.pdf]

File name: Supplementary Information
Description: Supplementary figures

File name: Peer Review File
Description:

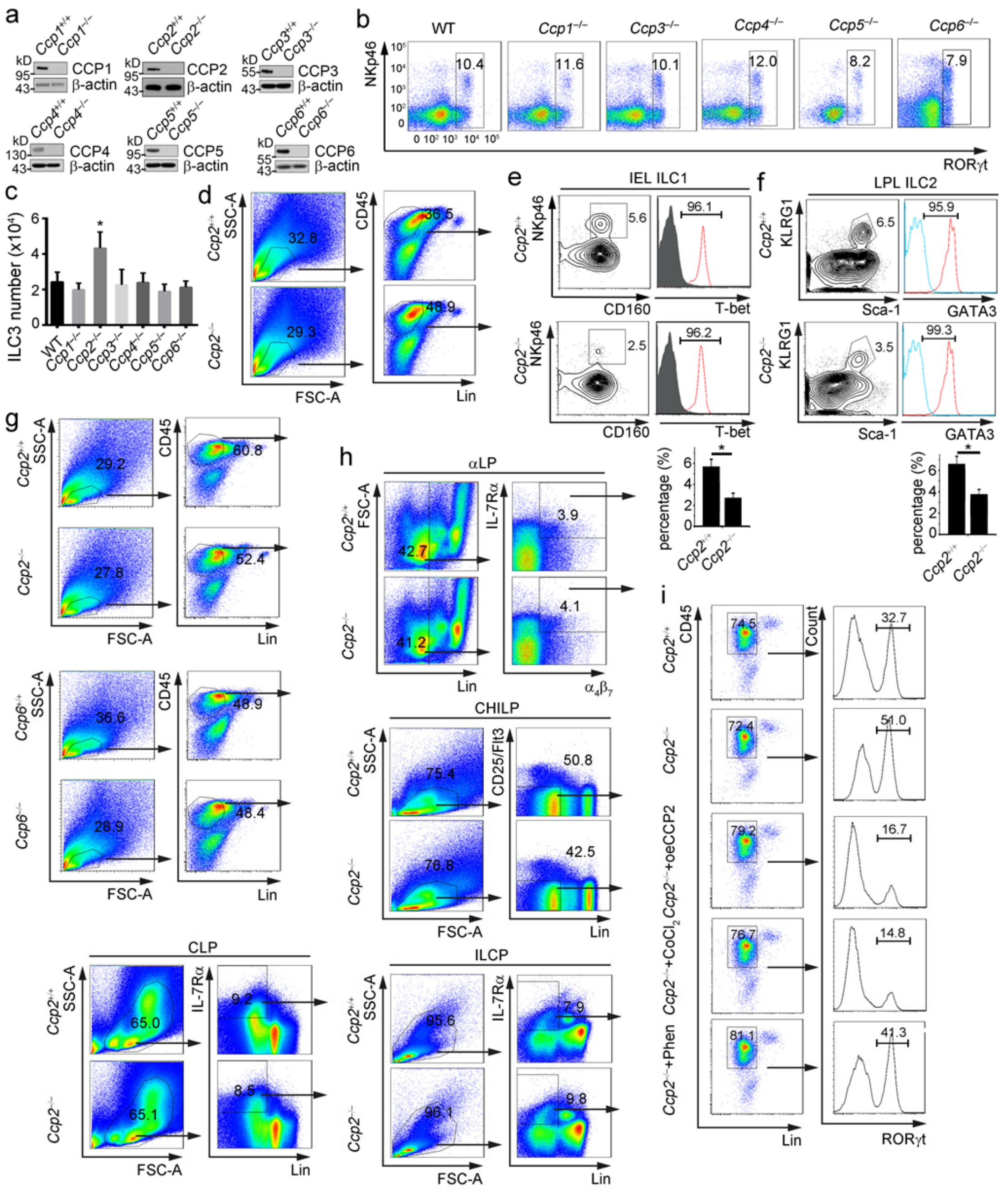

Supplementary Figure 1. Other CCP gene deficiencies have no impact on ILC3 development. (**a**) Deletion of *Ccp1*, *Ccp2*, *Ccp3*, *Ccp4*, *Ccp5* and *Ccp6* was confirmed in bone marrow by Western blotting. (**b**) Corresponds to data shown in Fig. 1a. Gating strategies and flow cytometry analysis of ILC3s in *Ccp1*, *Ccp3*, *Ccp4*, *Ccp5* and *Ccp6* knockout mice. n=6 for each group. Percentages of ILC3s were calculated. NS, no significant. ILC3 were gated out from $Lin^{-}CD45^{+}ROR\gamma t^{+}$ SI LPL cells. (**c**) Cell number calculations of ILC3 cells in *Ccp1*, *Ccp3*, *Ccp4*, *Ccp5* and *Ccp6* knockout mice. n=6 for each group. Data represent three independent experiments. (**d**) Corresponds to data shown in Fig. 1a and 1b. Gating strategies for gating out ILC3 from small intestines (SI). ILC3 were gated out from $Lin^{-}CD45^{+}ROR\gamma t^{+}$ SI LPL cells. (**e,f**) Gating strategy and flow cytometry analysis of ILC1s (**e**) and ILC2s (**f**) in small intestines from *Ccp2*$^{+/+}$ or *Ccp2*$^{-/-}$ mice. n=6 for each group. ILC1 cells were gated out from

$CD3^{-}CD19^{-}NK1.1^{+}IL\text{-}7R\alpha^{+}NKp46^{+}CD160^{+}T\text{-}bet^{+}$ SI IEL cells. ILC2 cells were gated out from $Lin^{-}CD25^{+}Sca\text{-}1^{+}KLRG1^{+}GATA3^{+}$ SI LPL cells. (**g**) Corresponds to data shown in Fig. 1d. Representative gating strategy of $IL\text{-}22^{+}$ ILC3 in SI LPL cells. (**h**) Corresponds to data shown in Fig. 2b. Representative gating strategy of CLPs, αLPs, CHILPs and ILCPs. (**i**) Corresponds to data shown in Fig. 2c and 2d. Representative gating strategy of $ROR\gamma t^{+}$ ILC3. Error bars in **c,e** and **f** indicate s.d.

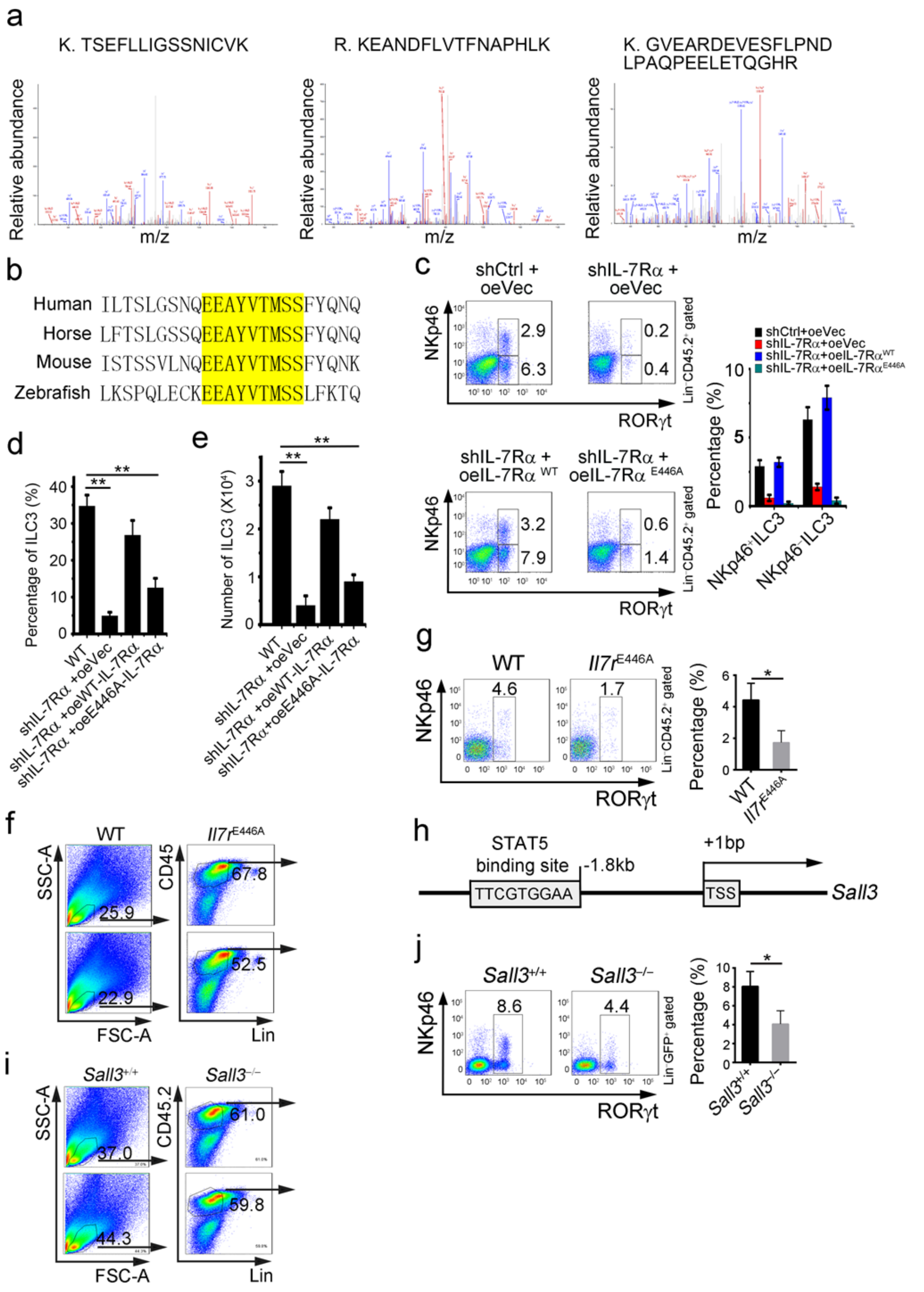

Supplementary Figure 2. IL-7Rα is a substrate of CCP2. (**a**) MS/MS profiles of IL-7Rα corresponding peptide sequences. (**b**) Amino acid alignment of IL-7Rα from different species. Conserved sequences are highlighted with yellow color. (**c**) 2×10^6 $CD45.2^+$ BM cells transfected with shIL-7Rα retrovirus and IL-7Rα-wt or IL-7Rα-E446A overexpression retrovirus were transplanted into lethally irradiated $CD45.1^+$ recipients for 8 weeks, followed by analysis of ILC3s. n=6 each group. (**d,e**) 2×10^3 CHILPs were

infected with shIL-7Rα retrovirus for silencing and then infected with WT-IL-7Rα or E446A-IL-7Rα overexpression retrovirus, followed by incubation with OP9 cells for 12 days. ILC3s were gated on $Lin^{-}CD45.2^{+}ROR\gamma t^{+}$. (**f**) Corresponds to data shown in Fig. 4j. Representative gating strategy of ILC3s. (**g**) 5×10^{4} $CD45.2^{+}$ LSKs from WT or *Il7r*^{E446A} mice with 5×10^{6} $CD45.1^{+}$ helper cells were transplanted into lethally irradiated $CD45.1^{+}$ recipients. After 8 weeks, percentages of ILC3s in chimeras were checked by FACS. n=6 for each group. (**h**) Analysis of STAT5 binding site on *Sall3* promoter. (**i**) Corresponds to data shown in Fig. 5n. Representative gating strategy of ILC3s. (**j**) 5×10^{4} GFP^{+} *Sall3*$^{+/+}$ or *Sall3*$^{-/-}$ LSKs with 5×10^{6} $CD45.1^{+}$ helper cells were transplanted into lethally irradiated $CD45.1^{+}$ recipients. After 8 weeks, percentages of ILC3s in chimeras were checked by FACS. GFP^{+} LSKs were isolated from *Sall3*$^{+/+}$ or *Sall3*$^{-/-}$ mice by FACS. n=6 for each group. $^{**}P<0.01$ (Student's *t*-test). Data are representative of three independent experiments. Error bars in **c**-**e**,**g** and **j** indicate s.d.

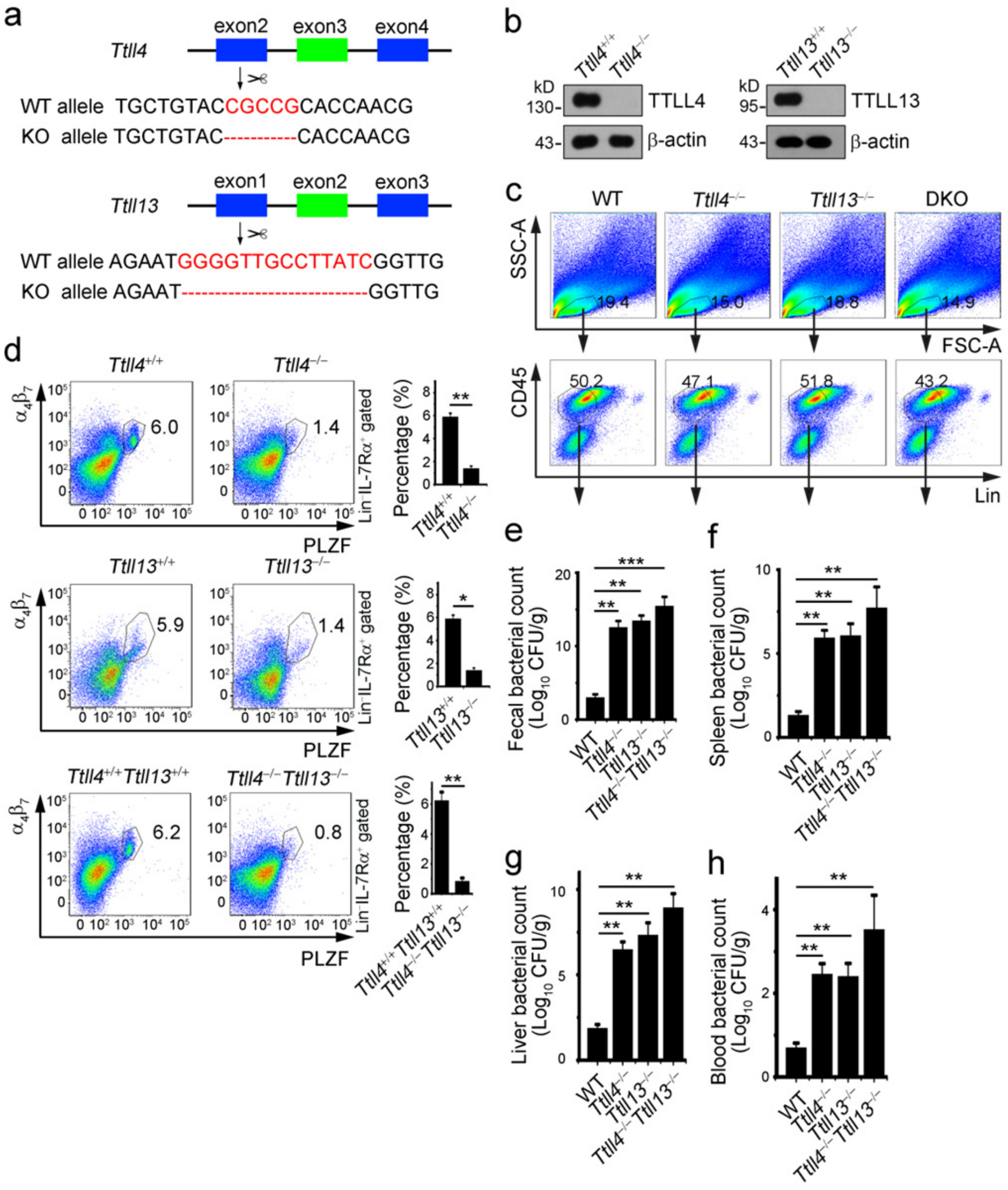

Supplementary Figure 3. Knockout mice of *Ttll4* or *Ttll13* are more susceptible to *C. rodentium* infection. (**a**) Knockout strategy for deletion of *Ttll4* and *Ttll13*. (**b**) TTLL4 or TTLL13 deletion was confirmed in bone marrow by western blotting. (**c**) Corresponds to data shown in Fig. 6d. Representative gating strategy of ILC3s. (**d**) Gating strategy and analysis of ILCPs from WT, *Ttll4*$^{-/-}$, *Ttll13*$^{-/-}$ and *Ttll4*$^{-/-}$*Ttll13*$^{-/-}$ mice by flow cytometry. ILCPs were gated on Lin^{-}IL-7R$\alpha^{+}\alpha_4\beta_7^{+}$PLZF^{+}. n=6 for each group. (**e-h**) Analysis of *C. rodentium* titers in fecals (**e**), spleens (**f**), livers (**g**) and blood (**h**) from WT, *Ttll4*$^{-/-}$, *Ttll13*$^{-/-}$, and *Ttll4*$^{-/-}$*Ttll13*$^{-/-}$ mice 8 d after *C. rodentium* infection. ***P*<0.01, ****P*<0.001 (Student's *t*-test). Data are representative of three independent experiments. Error bars in **d**–**h** indicate s.d.

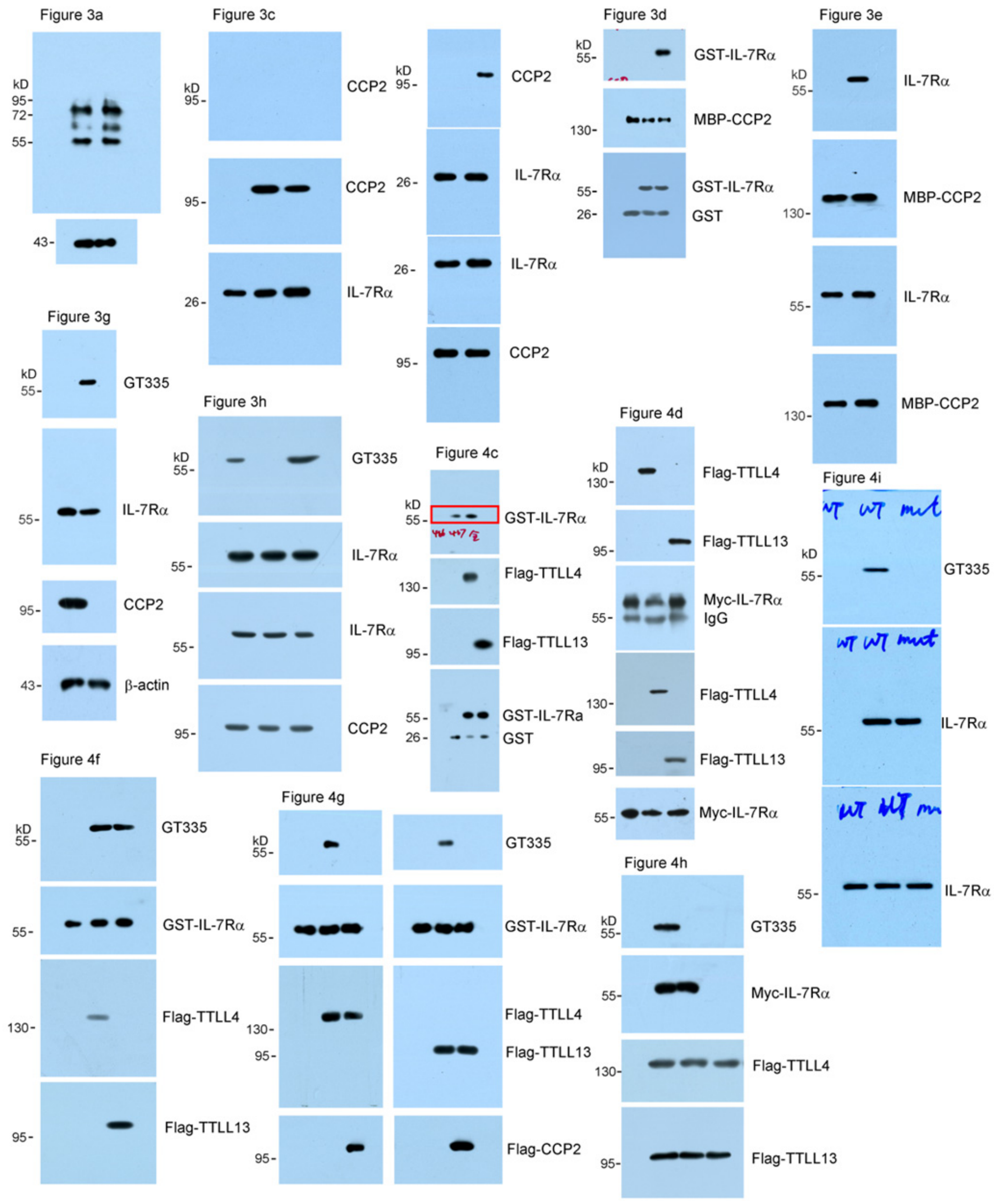

Supplementary Figure 4. Uncropped blots for Figure 3 and Figure 4.

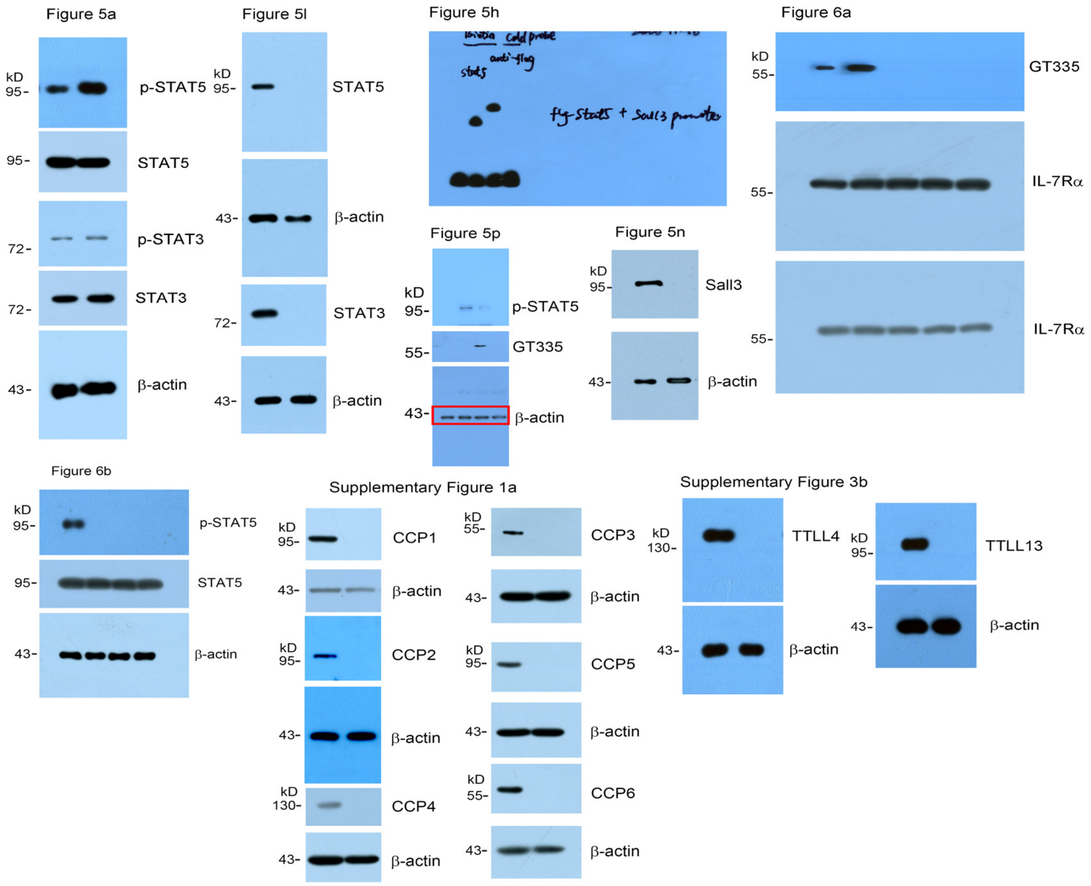

Supplementary Figure 5. Uncropped blots for Figure 6 and Supplementary Figure 1a and 3b.
